# Beyond Insoluble Dietary Fiber: Bioactive Compounds in Plant Foods

**DOI:** 10.3390/nu15194138

**Published:** 2023-09-25

**Authors:** Madeline Timm, Lisa C. Offringa, B. Jan-Willem Van Klinken, Joanne Slavin

**Affiliations:** 1 Department of Food Science and Nutrition, University of Minnesota—Twin Cities, 1334 Eckles Avenue, St. Paul, MN 55108, USA; timm0148@umn.edu; 2 Brightseed, 201 Haskins Way, San Francisco, CA 94080, USA; lisa.offringa@brightseedbio.com (L.C.O.); janwillem.vanklinken@brightseedbio.com (B.J.-W.V.K.)

**Keywords:** dietary fiber, bioactives, phytochemicals, plant foods, lignin, polyphenol, fruits, vegetables, health

## Abstract

Consumption of plant foods, including whole grains, vegetables, fruits, pulses, nuts, and seeds, is linked to improved health outcomes. Dietary fiber is a nutrient in plant foods that is associated with improved health outcomes, including a lower risk of chronic diseases such as cardiovascular disease, type 2 diabetes, and certain cancers. Different fibers deliver different health benefits based on their physiochemical properties (solubility, viscosity) and physiological effects (fermentability). Additionally, plant foods contain more than dietary fiber and are rich sources of bioactives, which also provide health benefits. The concept of the solubility of fiber was introduced in the 1970s as a method to explain physiological effects, an idea that is no longer accepted. Dividing total dietary fiber (TDF) into insoluble dietary fiber (IDF) and soluble dietary fiber (SDF) is an analytical distinction, and recent work finds that IDF intake is linked to a wide range of health benefits beyond increased stool weight. We have focused on the IDF content of plant foods and linked the concept of IDF to the bioactives in plant foods. Ancestral humans might have consumed as much as 100 g of dietary fiber daily, which also delivered bioactives that may be more important protective compounds in disease prevention. Isolating fibers to add to human diets may be of limited usefulness unless bioactives are included in the isolated fiber supplement.

## 1. Introduction

Plant foods and dietary fiber have been recommended in food guidance since 300 BC [1]. Plant foods contain more than just dietary fiber, so the protective properties of plant-based diets may be linked to other dietary components, including vitamins, minerals, or phytochemicals. Defining and measuring dietary fiber has always been challenging. From its start as crude fiber to its definition as dietary fiber, chemical methods have been developed and accepted as the standards for labeling and research purposes. Historically, fiber was considered to comprise polysaccharides (degree of polymerization [DP] > 10) that were resistant to digestion and absorption in the upper bowel and could then be fermented in the gut. The chemical composition of dietary fiber included cellulose, hemicellulose, pectin, and lignin [2]. The most abundant compounds identified as fiber are in the plant cell wall. Other fibers are part of the intracellular cement and others are secreted by the plant in response to injury. Human foodstuffs contain mainly noncellulosic polysaccharides, some cellulose, and little lignin. More recently, oligosaccharides, DP 3–9, have been included as dietary fiber in regulations and labeling, especially since these shorter-chain fibers are known to be prebiotic fibers. Overall, fruits and vegetables tend to be higher in cellulose than cereals. Lignin is highest in fruits with edible seeds (e.g., strawberries) or in mature vegetables such as carrots or other root vegetables. Dietary fiber composition of a plant depends upon plant species, maturity, and components (i.e., leaf, root, or stem). The human diet contains, in addition to polysaccharides and lignin, plant-derived materials similar to fiber that resist digestion in the small bowel, including cutin, waxes, and small amounts of proteins and lipids. Nonenzymatic browning products and nonhydrolyzable starch are often indigestible and therefore considered dietary fiber. 

Because the most widely accepted definition of dietary fiber is physiological, it is difficult to devise methods to measure dietary fiber and its components accurately. Historically, crude fiber was used, a method that treats food with acid and alkali to isolate the indigestible component. This method works for animal feeds, but it seriously underestimates the dietary fiber content of food, recovering only 50% to 80% of the cellulose, 10% to 50% of the lignin, and 20% of the hemicellulose [2]. Crude fiber values have no consistent relationship to the dietary fiber values of human foods; dietary fiber values are usually 3 to 5 times higher than crude fiber values, but no correction factors work because the relationship between crude fiber and dietary fiber varies depending on the plant. Bran flakes, for example, contain six times more dietary fiber than crude fiber, yet strawberries contain only 1.6 times more dietary fiber than crude fiber. The challenge of defining a simple, reproducible method to remove protein, fat, and soluble sugars and starch from food while retaining both water-soluble and insoluble components of dietary fiber continues to present challenges and limit work in dietary fiber.

### 1.1. A Shift from a Chemical Definition of Fiber to a Physiological Definition

Dietary fiber was first officially defined by the government in the United States in 2001 in an Institute of Medicine (IOM) document [3]. Dietary fiber is defined as nondigestible carbohydrates and lignin that are intrinsic and intact (i.e., naturally occurring) in plant-based foods. Thus, dietary fiber comes from the plant-based foods we eat: whole grains, vegetables, fruits, nuts, and pulses. Few plant foods are concentrated in fiber; popular plant foods in the US contain between 1 and 3 g of dietary fiber per serving [4]. Despite consumer perceptions that fruits and vegetables are loaded with fiber, because of their high water content, a serving of fruit contains at most 3 g of fiber, and most vegetable servings contain less than 3 g of dietary fiber. Grains, since they are commonly consumed, are the leading source of fiber in the diet. A serving of canned kidney beans provides 4.5 g of dietary fiber, but since legumes are not widely consumed, they are low contributors to fiber intake in the US. Since dietary fiber intakes are linked to calorie intakes, even with nutrient-dense food choices, it is difficult to reach the adequate intake (AI) of dietary fiber (28 g/day) with the recommended servings of plant foods. 

Isolated fiber can be added to foods and beverages or taken as bulk laxatives [5]. Consumers and healthcare professionals might not understand the complexity of dietary fiber, thinking it is a single nutrient in plant foods. Daily recommended intakes of fiber refer to total fiber without considering the source, type, quality, or physiological effects of the fiber. Although all added fibers contribute towards the recommended daily fiber intake, different isolated fibers deliver different health benefits based on their physiochemical properties (solubility, viscosity) and physiological effects (fermentability, bulking) [6]. 

Since the 1980s, the solubility of fiber has been promoted as an important attribute to explain the physiological differences among fiber sources. The solubility of fiber was thought to determine its physiological effects, with soluble fibers lowering blood lipids and blood glucose and insoluble fibers increasing stool weight and improving laxation [7]. It is now appreciated that the solubility of fiber has a minor role in determining physiological endpoints, and other factors, such as viscosity and fermentability, are key to the lipid-lowering effects of fiber, while fermentability and changes in the gut microbiota are more important physiological signals than the chemical properties of the fiber and whether it is soluble or insoluble [1]. Measuring total dietary fiber, soluble dietary fiber, and insoluble dietary fiber depends on fiber analytical methods that have evolved over time. For labeling purposes in the United States, total dietary fiber is required on the Nutrition Facts panel, while insoluble dietary fiber (IDF) and soluble dietary fiber (SDF) are optional. 

Support that soluble fibers lower cholesterol and insoluble fibers increase stool weight is lacking. Many fiber sources that are mostly soluble, such as oat bran and psyllium, still increase stool weight. Most fruits and vegetables are concentrated in insoluble fiber, not soluble fiber [8]. Cooked potatoes, oranges, and grapefruit are more concentrated soluble fiber sources [8]. Changes in fiber methods to measure total dietary fiber, soluble fiber, and insoluble fiber have evolved over the years, so agreeing on the amount of fiber in foods is challenging. 

Processing can either increase or decrease the fiber content of foods. Peeling fruits or vegetables generally decreases fiber content, although dietary fiber is also present in the pulp of fruits and seeds. Dried fruits will be higher in dietary fiber than fresh fruits, but this has to do with concentrating the fruit and taking out water. Blending fruits or vegetables will not decrease fiber content, as dietary fiber does not decrease with mechanical forces. Cooking also does not have a consistent effect on fiber content since if water is driven out in cooking, the total fiber content of a cooked product may be higher than the original product. Processing also does not significantly affect IDF or SDF in a predictable fashion. Although pulses are thought to be higher in SDF than IDF, many isolated proteins, such as soy and pea, are higher in IDF than SDF. Even in 2001, it was suggested that dividing SDF and IDF would not predict physiological response, yet consumers and health professionals continue to believe that SDF lowers blood lipids while IDF increases stool weight. 

### 1.2. Dietary Recommendations Are for Total Fiber, Not Soluble or Insoluble Fiber

The Institute of Medicine (IOM) set an adequate intake (AI) for dietary fiber of 14 g of fiber per 1000 kcals. This value is derived from data on the relationship between fiber consumption and coronary heart disease (CHD) risk. Dietary fiber is listed on the Nutrition Facts panel, and 28 g of dietary fiber is the daily value (DV) for a 2000 kcal diet. 

Despite the established benefits of dietary fiber, intake remains low. On average, the current intake is approximately half of the recommended levels, about 15 g/day [7]. The Dietary Guidelines for Americans currently classify dietary fiber as a “nutrient of concern”. While it has been established that fiber from intrinsic sources may be more beneficial to health, extrinsic sources via public health initiatives may be necessary [7]. Since extrinsic sources of isolated fibers are being added to foods and beverages, extrinsic IDF sources that also provide additional benefits like those from phytochemicals and bioactive compounds must be considered. Dietary fiber intake may be a proxy for plant food intake, and compounds beyond dietary fiber may provide important health benefits. Many of the phytochemicals are isolated as IDF, which makes it important to consider IDF as our best estimator of bioactive food components.

The National Institutes of Health (NIH) define bioactive food components “as constituents in food or dietary supplements, other than those needed to meet basic human nutritional needs, that are responsible for changes in health status”. Bioactive compounds are found in plant foods in differential quantities where, over time, they can contribute to health and wellbeing. These are not essential nutrients, like vitamin C, which is commonly measured and listed on food nutrition labels. An unlikely source of bioactive compounds is dietary fiber. These phytochemicals can be free and easily measured, or they can be bound in the fiber matrix and released during digestion. Measuring and reporting the bioactive content of foods, and specifically dietary fiber, is relatively uncommon but is becoming more mainstream with the recognition of bioactive compounds like curcumin in the food spice turmeric. 

### 1.3. Human Plant Foods and Protective Health Properties

Vegetarian diets have been promoted since the 18th century by men and women in search of physical and spiritual health [9]. A wide range of plant foods are consumed by humans, including most parts of the plant, fruits, seeds, leaves, roots, and tubers [10]. The nutrient content and potentially toxic constituents of plant foods vary greatly [11]. Generally, the concept of “moderation and variety” in food consumption limits the consumption of toxic amounts of compounds in plant foods, although work in the past has shown clearly that cooking and other forms of processing are important in removing toxic compounds in plant foods. As we continue to promote plant foods and dietary fiber, we must consider that all plants contain both protective and potentially toxic compounds and that the isolation process is critical in maintaining the compounds that have health benefits.

### 1.4. Whole Grains

Whole grains have been part of the human diet since the advent of agriculture about 10,000 years ago [1]. The invention of the roller mill in 1873 helped separate the bran and germ from the grain and provided refined grain foods that improved stability and consumer acceptance. Refined grains became popular, and enrichment and fortification policies focused on refined grains as a method to provide short-fall nutrients in the diet, such as B vitamins and iron, for example. Since refined grains are routinely consumed, fortification of nutrients continues to use refined grains as a vehicle to deliver necessary nutrients to vulnerable population groups. Since 1998, refined grains in the United States have been fortified with folic acid. 

Whole grains provide more than just dietary fiber, including vitamins, minerals, phytochemicals, and protein. The Dietary Guidelines for Americans recommend that half of your grains be whole, so three of the six grain servings should be whole. Less than 10% of Americans currently meet their whole grain intakes [1]. 

### 1.5. Fruits and Vegetables

Fruits and vegetables are universally promoted as healthy. Dietary Guidelines for Americans recommend you make one-half of your plate fruits and vegetables. Fruits and vegetables include a diverse group of plant foods that vary greatly in energy and nutrients [8]. Classification of fruit and vegetable exposure in epidemiologic studies is challenging as there is no accepted definition [12]. Culinary custom is often used instead of botanical definitions. Botanical fruits include squash, tomatoes, and mature beans, while these are culinary vegetables [12]. Nutrients and phytochemicals are often concentrated in certain categories, i.e., vitamin C in citrus fruits, carotenoids in orange vegetables, etc. Fruits and vegetables provide more than dietary fiber and are rich in bioactives, although compositional data on the wide range of bioactives in fruits and vegetables is difficult to access in the literature. While the dietary fiber content of fruits and vegetables is listed in nutrient databases, the bioactive composition of popular fruits and vegetables is not.

Fruits and vegetables can also be raw, cooked, made into juice, pickled, dried, or eaten in mixed dishes. Even what counts as a serving of fruit or vegetable continues to be debated [8]. Particularly challenging is whether juice counts as a fruit serving or whether high-fat or high-sodium vegetables count, for example, French fries or potato chips. Fruit juices with pulp will contain dietary fiber and are likely to contain bioactives in the whole fruit. Dietary recommendations for fruit consumption are based on obtaining the nutrients found in fruits: vitamin C, vitamin A, potassium, and dietary fiber. Vegetables vary greatly, whether leafy green vegetables or tubers. Vegetables provide a range of nutrients but additionally contain protein and starch, if they are root vegetables. 

Recommendations to increase consumption of fruits and vegetables remain based on the nutrients that these foods contain, such as dietary fiber, vitamin C, vitamin A, and potassium, rather than the phytochemicals or bioactive compounds that are provided by fruits and vegetables. Fruits and vegetables are routinely underconsumed by consumers, resulting in low intakes of dietary fiber, potassium, and other nutrients. The bioactives that are concentrated in different vegetables and fruits are also underconsumed when consumers choose diets low in fruits and vegetables.

### 1.6. Legumes/Pulses

Pulses are a dry, edible variety of beans, peas, and lentils that are rich in plant-based protein and fiber, as well as micronutrients such as iron and potassium [13]. Pulses can count as either a vegetable or a protein source on myplate.gov. Much of the analytical information on the fiber content of pulses is based on the uncooked variety. Dry beans, lentils, and peas must list the dietary fiber content that is on the package, not the amount of dietary fiber that is provided in a cooked portion of the legume. Pulses are usually promoted as a concentrated source of SDF, while many are actually richer sources of IDF.

### 1.7. Regulations on Insoluble Fiber

The FDA published a final rule in 2016 that created a definition for dietary fiber: “non-digestible soluble and insoluble carbohydrates (with 3 or more monomeric units), and lignin that are intrinsic and intact in plants; isolated or synthetic non-digestible carbohydrates (with 3 or more monomeric units) determined by FDA to have physiological effects that are beneficial to human health” [21 C.F.R. 101.9(c)(6)(i)] [14]. The category of “intrinsic and intact plant fibers” occurs in vegetables, whole grains, fruits, cereals, and flours. Intact refers to the fact that the fiber was not removed from the food. These are mostly—but not limited to—insoluble fibers. The other category of fiber is removed from their original plant food or derived synthetically and is called functional fibers. New FDA regulations require that these isolated fibers show a “beneficial physiological effect” before they are accepted as dietary fiber by the FDA. These isolated fibers still need to be listed on the ingredient list, but they cannot claim any dietary fiber on the Nutrition Facts panel unless they have been accepted by the FDA as having a physiological benefit.

### 1.8. Purpose/Aim

The purpose of this review is to investigate the nutritive value of insoluble dietary fiber (IDF) fractions with respect to their rich bioactive content. The mass production of some goods yields high amounts of byproducts, including fruit peels and pomaces, that are rich in insoluble fiber but also rich in bioactive compounds. Plant byproducts are a potential nutrient-rich option to address low fiber intakes with minimal risk of adverse effects. These byproducts are commonly discarded, composted, used as animal feed, or used in the beauty industry. But the volume of the byproducts is large enough to be disruptive to the environment [15]. Using them in the food industry will reduce that volume and can increase both fiber and bioactive compound intake when consumed in food. These products could also be attractive to customers who seek out food products with additional health benefits [15]. 

### 1.9. Key Questions (Figure 1)

1. What IDF sources have been examined in regard to their bioactive content?
2. What bioactive compounds are present in IDF?
3. Do these bioactive compounds exhibit health-promoting effects?

**Figure 1:**
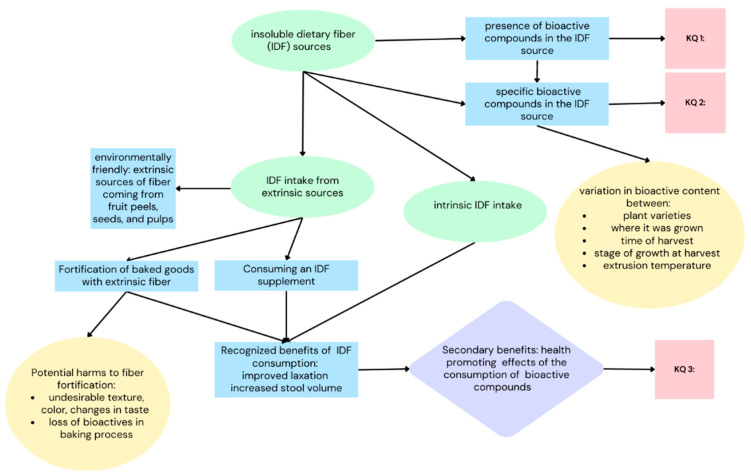
Conceptual model depicting the key questions framing the review.

## 2. Materials and Methods

### 2.1. Search Strategy

To conduct the scoping review, three databases were used to search for relevant research: Ovid Medline, Ovid Agricola, and Scopus. These three databases were chosen to encompass a variety of fiber and bioactive research. Current research was observed in both medical and agricultural journals, hence the inclusion of Medline/Scopus and Agricola. The Institute for Food and Nutrition Sciences (IAFANS) dietary fiber database was also screened. It was ultimately excluded due to its lack of research regarding phytochemicals in fiber and its focus on glycemic control, hunger/fullness, and type II diabetes. The search string was altered to fit the requirements of the respective databases. Ovid allowed for the same search string to screen two databases simultaneously, Medline and Agricola. For Scopus, the string had to be altered slightly to adhere to their format. The search string can be found in Table 1. No hand-searching was utilized. The insoluble fiber types initially included in the search were chosen on the basis of their current availability on the commercial market and can also be found in Table 1. But this was not a limiting factor, as other fiber types were added to the string before finalization if they appeared in the initial exploration of literature. Other insoluble dietary fiber types appeared in the search results, and they were not excluded due to the nature of the scoping review. 

### 2.2. Study Selection Process

**Figure 2:**
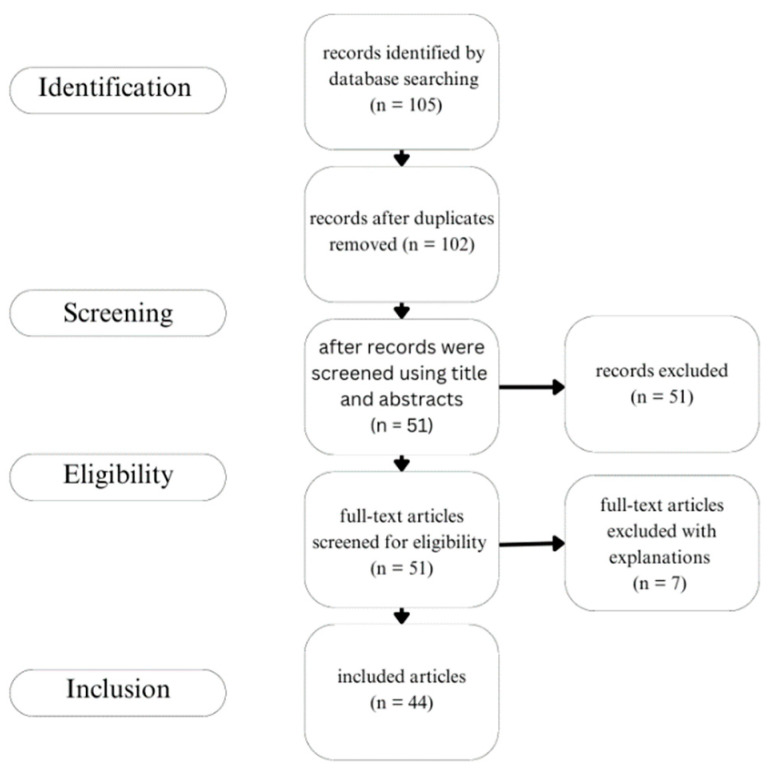
Results of the literature search and screen.

The search string yielded a total of 105 articles: 98 from Scopus and 7 from Ovid Medline and Ovid Agricola (Figure 2). After the duplicates were removed 102 articles were left. One individual conducted the study selection process. The screening entailed reading the title and abstract of each article and determining whether they were to be excluded or eligible for the full-text screening. Articles were removed if they were unrelated to the topic or if they did not examine the bioactivity of the fiber source/removed the fiber from the source prior to the experiment. The study selection process was limited by multiple additional factors. In terms of study characteristics, the study had to be originally published in English. The articles also had to be primary literature; all reviews were excluded. There was no recency requirement placed on the study selection process. The majority of the articles were published during or after 2011. There was one outlier that was published in 1997. At the eligibility stage, the remaining articles (*n* = 51) were screened for their eligibility by reading the full text. After full-text screening, 44 articles remained, with 7 additional articles being excluded (Figure 2). The study selection results can be found in Table 2, and the list of excluded articles after the full-text screen can be found in Appendix A. 

## 3. Results

### 3.1. Bioactive Sources in Insoluble Dietary Fiber

A variety of insoluble fiber sources appeared in the literature search. The search string included 17 sources, and the results yielded 30 IDF sources. The 17 sources that were part of the original search were: apple, beet, blueberry, carob, carrot, chickpea, citrus, cranberry, kiwi, pea, potato, chia, flax, hemp, corn, oats, rice, soy, and wheat. The 30 fiber sources evaluated for bioactive content were: rice, soybean, oat, corn, wheat, carob, cashew, chickpea, linseed, lentils, mango, tomato, persimmon, beets, apple, swiss chard, pea, pear, orange, pineapple, guava, cherry, pitaya, capuassu, pomegranate, plum, grape, sapodilla, eggplant, carrot, gourd, mentha, spinach, ginseng, blueberry, raspberry, cranberry, lemon, carambola, watermelon, and stinging nettle. The bioactive content was evaluated by three methods: total phenolic content (TPC), total flavonoid content (TFC), and antioxidant activity (AA). Not every source was evaluated with all three methods. Table 2 depicts the fiber type and whether TPC, TFC, and/or AA were used to evaluate the source. TPC was primarily conducted using the Folin–Ciocalteu reagent and were expressed in the units of mg GAE/g and mg/g gallic acid. TFC was typically measured by an absorbance assay and expressed in either mg quercetin equivalents/g or mg rutin/g. An additional note is that polyphenols are flavonoids because of their multiple ring structure, so TFC is a subset of TPC. 

When AA was measured, it was conducted in a variety of ways. Methods that appeared in the search results can be depicted in Table 3. They included free radical scavenging assays (ABTS and DPPH) and ferric reducing antioxidant power assays (FRAP) most frequently. Other methods that were utilized were cellular antioxidant activity (CAA), Trolox equivalent antioxidant assay (TEAC), beta-carotene bleaching assay, photochemiluminescence assay, and PCL. 

### 3.2. Bioactive Compounds in Sources of IDF

There were a total of sixty-four bioactive compounds detected across the insoluble fiber sources that appeared in the research. They fell into three categories: phenolic acids, flavonoids, and non-flavonoid compounds. The non-flavonoid compounds included tannins, tocopherols, tocotrienols, vitamin C, retinol equivalents, carotenoids, chlorophylls, and betalains. The majority of these were detected using high-performance liquid chromatography (HPLC). The specific compounds can be found in Table 4.

## 4. Results and Discussion

### 4.1. Sources of Insoluble Dietary Fiber and Bioactives

In addition to bioactive flavonoids and phenolics, IDF can be found specifically in certain plant foods but also in different tissues within these plants. Guava (Table 3) contains phenolics and flavonoids, which are a class of phenolics, and has a high proportion of insoluble dietary fiber (11.81 g/100 g). Many plants contain tissues that have different types of fiber, or one tissue type will contain fiber where the other does not. Fruits like apples have less insoluble dietary fiber (1.54 g/100 g) because most of the insoluble fiber in an apple is in the peel, not the pulp [57]. Seeds are used for oil and protein, but the seed coat, or hull, contains dietary fiber. For example, hemp seed hulls contain 46% cellulose, 31% lignin, and 22% hemicellulose, which are all types of insoluble dietary fiber [58]. Of the total seed, 22.25% is insoluble dietary fiber [59]. In addition to IDF, hemp hulls contain lignanamides, including cannabisin F-type (34–50 mg/100 g DW) and grossamide-type (76–292 mg/100 g DW) [32]. These bioactive compounds are formed using hydroxycinnamic acid amides as intermediates and have in vitro antioxidant and anti-inflammatory activity [60,61]. Blood orange fruits are rich in bioactives; seeds were the highest in limonoids, but peels had the highest flavonoid content. The pulp had the highest TDF and a large IDF-to-SDF ratio compared to juice, seeds, peels, waste water, and solid residues [51]. Bioactivity was high across the whole fruit, but the specific compound content varied based on what part of the fruit was utilized. 

### 4.2. TPC, TFC, and AA

Overall, the studies that tested TPC found that there was an increase with the addition of IDF fractions. Fortification of IDF into a baked good did decrease TPC in comparison to the pure fraction, but the bioactive content still remained higher than the control flours. This can be expected due to the cooking process and using a combination of the IDF fraction flour and the control flour in an effort to maintain the integrity of the baked goods. In comparing the TPC of oat milk byproduct (OMB) flour and wheat flour, OMB flour had a higher TPC [22]. And when the OMB flour was fortified with chickpea flour, the TPC increased further. 

Total flavonoid content was high as well across multiple IDF sources. IDF fractions that were made from peels tended to exhibit increased levels of flavonoids. The production of flavonoids is thought to be linked to exposure to sunlight [33]. When comparing flour fortified with mango peel and mango pulp, mango peel flours, made from ripe and green mangos, had higher flavonoid contents [35]. But both mango flours had higher bioactive contents compared to pure wheat flour. 

The AA assays provide a general overview of the potential bioactivity of food products. Similarly, for TPC and TFC, there were increases in AA with the IDF fractions compared to the control, typically white or wheat flour. OMB had significantly higher AA on FRAP and DPPH assays compared to wheat flour [22]. These increases mean that there is more AA, but it does not reveal any information about what antioxidants are present, but it does indicate that there is some kind of elevated nutritive value to the IDF. 

### 4.3. Extraction, Processing, and Maintenance

The extraction and processing of IDF fractions have significant effects on the bioactive and IDF content of the said fraction. Sieve size was one factor that yielded changes in bioactivity. When using multiple sieve sizes to smooth out dried beetroot leaves powder (DLBP), Asadi et al. found that smaller sieve sizes had lower levels of IDF. They observed the highest levels of IDF in the raw DBLP [39]. Similar results were observed with other fiber sources. As the sieve size decreased, the IDF content of pitaya peel powder (PPP) decreased and SDF increased [15]. This is thought to be due to the force pushing the PPP through the sieve; the IDF was transformed into SDF because the sieve broke the bonds between hemicellulose and lignan. But decreasing sieve size did not significantly alter the bioactive content of the PPP; AA and TPC remained relatively consistent between sieve sizes, and betacyanin content was significantly consistent between the 4 sizes [15]. 

Treatment of the IDF sources also impacted bioactivity. Lau et al. used a conventional extraction method and a supercritical fluid extraction (SFE) method to acquire carotenoids from sweet corncobs [24]. They observed that the SFE method extracted more carotenoids than the traditional method. Similar results were seen in the extraction of fiber from Mexican apple pomace powders. When the fiber was extracted with methanol, the phenolic content was much higher than the samples extracted with methanol and acetone [43]. Pretreatment also played a role. In 2011, an alkaline treatment was used on raw corncob in an effort to decrease the silica content. And while the silica and oil content of the corncob decreased, so did AA, measured via DPPH. In 2012, a similar study was conducted where alkaline treatment was used to decrease the silica content of rice husk and decreased AA via DPPH, and a decrease in mineral content was observed [19]. They concluded that the separation of soluble and insoluble residues may not be helpful in an effort to increase the nutritive qualities of food. In this case, extracting just the insoluble residues resulted in a loss of minerals, which led to a reduction in AA, suggesting that being a food source rich in insoluble fiber may be more beneficial than being purely insoluble fiber. The bioavailability of the phenolics possibly being affected by the extraction method is also important. Phenolics can be in a bound form or a free form, and the bound phenolics are considered to be less bioavailable. The insoluble, soluble, and total fiber rice bran fractions were examined for phenolic content and AA, and the insoluble fraction had the highest levels of bound phenolics and CAA activity, but it had the lowest phenolic activity due to the inaccessibility of the bound phenolics [18]. Total rice bran and the soluble fiber fraction had the highest levels of phenolic activity, indicating that the use of rice bran as an IDF source may be more valuable in its whole form rather than undergoing extraction, similar to what Kuan et al. concluded in an effort to separate the insoluble fiber from rice [19]. 

Extrusion temperature was another factor that played a role in bioactivity. The purification of cactus peel powders via increased temperature processing yielded decreases in TPC [48]. Medeiros et al. observed the same results, seeing decreases in TPC of fruit pomaces upon thermal processing [28]. Although TPC decreased, AA remained above 70% of the activity measured before thermal processing [28]. This indicated that the fruit pomaces were still considerably bioactive despite the temperature. Although mango pulp fiber waste (MPFW) was dried, the total flavonoid content decreased by 50% [34]. One study indicated that the size of the IDF fraction may be more impactful than the extrusion temperature. When rice flour for gluten-free bread was extruded at 120 degrees C instead of 80 degrees C, a higher phenolic content was observed when there was a higher fraction of sour cherry pomace added to the bread [53]. So, higher temperatures and a higher IDF fraction yielded higher phenolic contents in comparison to rice flour extruded at lower temperatures. This is useful for the future of fortifying baked goods with IDF fractions, because when fortifying a good with IDF extrusion, temperature may not be as important in the maintenance of bioactivity. Another aspect of extrusion temperature that is useful is consumer acceptability. Butterfly pea flower (BPF) was added to puffed cereal, and consumers preferred the cereal extruded at a higher temperature because it was crispier [47]. While the cereal was crispier, the phenolic content decreased, but as the percentage of BPF increased, so did the ferric reducing power and AA, measured via DPPH. And there were no differences in extrusion temperature—130 degrees C versus 150 degrees C. Bioactivity and acceptability increased with higher extrusion temperatures at the cost of a decreased TPC. In conclusion, temperature does affect bioactive content, but it does so differently depending on the fiber source. 

Outside of the extraction and processing method of the fiber fraction, the maintenance of bioactivity is another consideration. Like temperature during processing, temperature during cooking alters bioactivity. After boiling lentil-fortified pasta, 30% of the phenolic content was lost [31]. And when boiling gluten-free pasta enriched with tomato waste and linseed meal, a loss of phenolic compounds was observed [29]. The fortification of flour, respectively, with black, blue, and purple wheats increased anthocyanin and insoluble phenolic content, which then decreased after cooking of chapatti [26]. Despite the decrease in bioactivity following cooking, all of the goods retained a level of bioactivity that was higher than when the goods were made with the control flour and not fortified. Another example of this was demonstrated in the development of ginseng residue (GSI)-supplemented bread. Pure GSI flour had nine bioactive compounds, whereas the control flour had none. The bread was supplemented with 2, 4, 6, and 8% GSI flour, and not all of the baked breads had bioactive compounds, but all of them had syringic acid, epicatechin, p-coumaric acid, and ferulic acid, which is more than the wheat flour control [55]. And as the percentage of GSI increased, so did the number of bioactive compounds; 8% of GSI bread was detected. So, while the compounds were not fully retained, there was still an improvement in nutritive value outside of an increased amount of IDF. 

### 4.4. Food Applications

The bioactivity and high IDF content of these plant fiber sources present an opportunity for fortification. Ready-to-consume food products are largely baked goods and have a low nutritive value overall. When plant sources were added to cookies, the IDF, TPC, and TFC content of the cookies increased. This occurred with increasing fractions of DBLP [39]. As the sieve size decreased, IDF decreased, but if the PPP was not sieved, there was an increase in IDF in the cookie [15]. Both cookies also had a decreased amount of carbohydrates. The addition of OMB to cookies increased the IDF, and the addition of chickpea flour further increased the IDF, but chickpea flour decreased the beta-glucan content and TFC of the cookies and increased the carbohydrates [22]. Chickpea flour was added to the good because the pure OMB flour gave the baked good a bitter flavor. 

Other goods were fortified, one being muffins with mango pulp flour. The MPFW and dried mango pulp fiber waste (DMPFW) muffins had higher beta-carotene and lutein contents compared to the control muffins with wheat flour [34]. Outside of being fortified, pure MPFW and DMPFW were rich in phenolics, but when both were in muffins, only gallic acid, catechin, and epicatechin were detected [34]. Bread was also fortified with a variety of plant foods: ginseng residue, stabilized rice bran (SRB), sour cherry pomace, and colored wheats. The addition of tomato pomace to crackers increased the IDF, SDF, TDF, protein, and mineral content, and acceptability scores were consistent between 0, 4, and 8% for tomato pomace [38]. Noodles and cereal grains were fortified as well. The addition of stinging nettle increased chlorophyll and carotenoid content, and the addition of tomato pomace and linseed meal increased the tocol content of the noodles but not the carotenoid content [29,56]. When tomato pomace and lentils were added to tarhana, there was an increase in IDF, AA, and TPC [30,38]. The same three measures of bioactivity increased with the addition of teff to gluten-free breakfast cereals, but crispiness, porosity, and lightness decreased [23]. The addition of IDF plant sources to yogurt was also carried out. Okara, a waste product from soybean processing, had high levels of AA and TPC. It was not successfully added to yogurt due to undesirable textures and a shortened shelf life, but its nutritive content indicates that it may be a potential additive to other products or used as a supplement [21].

The addition of insoluble fibers was also shown to be a functional additive to products. Apple pomace (AP) was used in an effort to maintain the integrity of yogurt. Over 28 days, they concluded that 0.5% (AP) yielded a yogurt that was firmer, more consistent, and had a higher IDF than the control [45]. Sugar beet pectin (SBP) was used as a carrier for curcumin to prevent degradation of the nutrient and extend the shelf life of the product it was added to. The unprotected degradation of curcumin was 40% after 4 days and 45% over 15 days, but upon the addition of curcumin, the protected degradation was 8% and 23% over the same time frames [40]. Bioactive compounds were observed in beetroot powders, including saponins, terpenoids, flavonoids, and phytosterols [41]. 

In conclusion, the addition of IDF to food increases its bioactive content. When small amounts of IDF are added to the flour used to create a baked good, AA and TPC increase without compromising the integrity of the good. Acceptability scores were high as well. Bioactivity did decrease upon cooking, but it still remained higher than the control food, and it may be useful as a supplement for consumers.

### 4.5. Factors That Influence Bioactivity

Bioactive content was influenced by multiple factors outside of extraction, processing, and maintenance. Between five different apple varieties, IDF, TPC, and AA varied, with Royal Delicious having the highest for all three measurements [44]. Three colored wheats had different anthocyanin contents, despite being the same plant [26]. In rice varieties, ferulic and p-coumaric acid predominated in the KFSW variety, while ferulic acid, p-coumaric acid, and caffeic acid were the major phenolics in the TK16 variety [20].

Differences in harvesting time in terms of year of growth, time of year, and time in the growth process also influenced bioactive content. When wheat grain was harvested in two consecutive years, water-soluble and total arabinoxylans were not detected in the first year, but they were detected in the second [25]. The opposite observation was made when carotenoid content was measured in persimmon fruits. In 2017, neoxanthin, violaxanthin, retinol equivalents, and beta-cryptoxanthin were present, but almost none were detected in 2018. Only one sample had neoxanthin, and at a much lower level. There were also variations based on the time of year; stinging nettle was harvested in April and May because research had previously shown that harvesting at that time yielded a plant that had a higher mineral content [56]. Bioactivity also varied based on when the plant was harvested during its growth process. Immature rice grains had two times the amount of total tocols as mature rice grains in both KFSW and TK16 varieties. But the gamma-oryzanol content did not significantly differ between mature and immature rice grains [20]. Lin et al. concluded that immature rice grains are a potential nutraceutical. Green and ripe mango peels had higher IDF, SDF, and vitamin C content than the wheat flours. The green mango peels had higher levels of TPC and ascorbic acid, whereas the ripe peels had higher levels of anthocyanins [35]. These results were consistent with the study conducted by Lin et al. [20], which found that immature plants may be richer in bioactives than their ripe counterparts.

The fraction of the fiber source used influenced bioactivity as well. The soluble and insoluble components of defatted rice bran (DRB) were examined, as well as the intact fraction of DRB. The intact DRB had more bioactive compounds than its counterparts, respectively, indicating that the entire plant may be more beneficial as a nutraceutical. Insoluble DRB had a higher proportion of bound phenolics and AA activity, but the phenolics were measured to be less bio-accessible [18]. It is unclear whether or not the digestion process increases accessibility.

### 4.6. Health Benefits of Bioactive Compounds

Bioactive compounds have a variety of health benefits. Dietary polyphenol consumption is associated with the management of free radicals and reactive oxygen species [44]. The IDF and bioactive-rich plant sources were associated with the management of blood glucose. The addition of stinging nettle to wheat durum pasta reduced the glycemic index (GI) when added in 1, 2, and 3% amounts [56]. The glycemic index was the smallest with the 3% stinging nettle addition to the pasta. While wheat durum pasta already has a relatively low GI index, it lowered even further. The addition of GSI residue also reduced the GI index of bread, starting at a 2% addition, and as the fraction increased, the GI index continued to decrease. The addition of GSI also increased the cholesterol absorption capacity; this is likely due to an increase in IDF [55]. Mango pulp fiber had a balanced ratio of IDF to SDF, which is an indication of a low GI index [34]. Stinging nettle, GSI, and mango pulp fibers are potential options for the management of blood glucose; this can be especially beneficial for those who have or are at risk for type II diabetes.

Alpha-amylase activity is another measure of blood glucose control. The alpha-amylase enzyme slows the metabolism of glucose by limiting its digestibility and absorption ability in the GI tract, which results in lower postprandial blood glucose levels. Blue grapes exhibited a high alpha-amylase activity that was hypothesized to be due to the high tannin content of the fruit [50]. Wild swiss chard leaves were identified as a possible anti-diabetic medication due to their alpha-amylase and alpha-glucosidase activity [46]. And the alpha-amylase activity of pineapple pomace was comparable to cellulose [49]. The beta-glucan content of these bioactively rich IDF sources also increased in comparison to the control. Adding oat milk byproducts to biscuits traditionally made with wheat flour increased the beta-glucan content. This has been linked to an increase in the water absorption capacity (increased freshness) but also a decreased risk of hypertension, obesity, and diabetes [22]. The total lactic acid bacteria (LAB) content was increased in lentil flour compared to wheat flour, which indicates a possible benefit to gastrointestinal health [30]. The scope of this review is not to provide an overview of all the potential benefits of the reported bioactive compounds. If additional information is desired, it is recommended that the reader refer to other articles.

### 4.7. Applicability

This research is applicable to a wide variety of consumers. Focus groups were conducted in many of the studies that fortified a baked good. These are valuable because even if the nutritive content of the good increases, if it is not palatable, consumers will not purchase or consume it. And if it is not consumed, the benefits upon consumption are going to be lost. The range of ages that participated in acceptance testing ranged from college students to middle-/late-aged adults. The majority of the focus groups utilized hedonic scales that had the participants rank characteristics like color, aroma, texture, flavor, softness, and overall likeness on a scale from “extremely dislike” to extremely like” and utilized untrained or trained participants. There was no significant difference in acceptability scores between breads fortified with 0, 3, and 6% capuassu peel powder [54]. And there were no significant decreases in sensory qualities recorded after the addition of stinging nettle to wheat durum pasta [56]. These results indicate that the utilization of fortification at a low level does not significantly alter the acceptability of the product to the consumer while still increasing its nutritive value via IDF and bioactive compounds.

The addition of IDF plant sources to ready-to-eat products also presents an option for gluten-free individuals to increase their DF intake, as it is typically a nutrient of concern for that population [53]. Fiber typically comes from whole grains, fruits, and vegetables, where whole grains are the best source and fruits and vegetables need to be consumed in large amounts to reach an adequate intake. Fortifying a rice-based gluten-free bread with sour cherry pomace and increased soluble fiber, insoluble fiber, TPC, and antioxidant content in breads that were made with rice flours extruded at 120 degrees C [53]. When oat milk byproduct (OMB) was added to biscuits, there were increases in protein content, dietary fiber, beta-glucan, total polyphenol content (TPC), and total antioxidant capacity [22]. This is beneficial not only to those who have celiac disease but also to the general population as the number of people electively choosing gluten-free diets increases.

Plant sources of IDF and bioactives are cost-effective and environmentally friendly, as the majority of them are byproducts of commercial food processing. While food waste can be composted, too much compost can overload an ecosystem and, in turn, have negative environmental effects. These byproducts are also cost-effective because they are already being discarded. They can also be obtained without shipping long distances because they are present in a variety of products. For example, there is high sour cherry juice production in Poland, resulting in a lot of sour cherry pomace waste [53]. Pitaya peel is more easily available in tropical and subtropical regions, and capuassu is available in Brazil [15,54]. There are other sources of bioactive-rich IDF that are still environmentally friendly while not relying on another industry, like stinging nettle and wild edible Swiss chard [46,56].

### 4.8. Research Recommendations/Future

In the future, more research needs to be conducted in this area. There is potential to increase the nutritive value of food. In considering the production of a supplement or powder to use for fortification, researchers will need to consider the fiber type, season and climate of harvest, ripeness at harvest, and more to maximize the bioactive content of their product. They will then need to investigate the degradation over time. Does the byproduct need to be used right after it is harvested? And can it maintain its integrity in a product with a long shelf life? Or will it alter the shelf life of the good it is incorporated into? These are especially important for the bioactives that are known to be susceptible to degradation by light exposure or those not soluble in liquid. They should also consider and research the effects of enzymatic pre-treatment as a method of preparing the IDF fraction for storage.

Consumer acceptance is also an important aspect of this research. While it is important to successfully obtain an IDF fraction rich in bioactives, if it is not acceptable to the consumer, that will not improve health. Some customers may not try a cookie if they know tomato is in it or bread that looks pink because the addition of sour cherry pomace makes it look different [37,53]. But at the same time, many consumers are attracted to those products that have additional health benefits or are considered natural because the benefits outweigh the altered texture, taste, etc.

Future research needs to consider the phytic acid content of plants as well. This is particularly important in the use of rice but can be applied to other plants, including grains, seeds, legumes, and tubers. Phytic acid is a chelator that can result in mineral deficiencies, especially zinc. Leavening agents can break down phytic acid and release minerals for absorption, so the processing effects will determine if phytic acid has any negative effects in the diet. When capuassu peel powder was added to bread, total tannins and total phenolics increased, but so did the phytic acid content [54]. The use of pre-treatment is an option to manage phytic acid content. Stabilizing rice bran decreased the phytic acid content and prevented the rice from going rancid, and the germination of rice also decreased the phytic acid content [16,17].

## 5. Conclusions

In conclusion, insoluble dietary fiber is rich in bioactive compounds. It has the potential to be a nutritive product in the form of a supplement or fortified into a food product. Multiple considerations must be made regarding the extraction, processing, and storage of these bioactive compounds, but the majority observed an increase in bioactive content, even with the addition of a small amount of IDF. Work on whole grains has suggested that the bioactives in whole grains may be more important than the dietary fiber in the grain. Considerations on how best to maintain the bioactive compounds in plant extracts while still maintaining the fiber content are needed to increase both dietary fiber intake and the intake of bioactives. Past thinking that soluble dietary fiber has the most physiological benefits while insoluble fiber only alters bowel function is no longer accepted. Dietary guidance must continue to support increased consumption of plant foods to increase our total dietary fiber intake to the recommended levels. The health-promoting effects of insoluble dietary fiber go beyond just dietary fiber, and future efforts to isolate fibers from plant foods need to consider bioactives and processing strategies to enhance both dietary fiber and the bioactive content of the supplement.

## Figures and Tables

**Table 1:** Search string for insoluble fiber types and bioactive content.

Database	Search String
Ovid Medline/Ovid Agricola	[insoluble fiber.mp.] AND [“phytochemical.mp. or Phytochemicals/” OR bioactive.mp. OR phytonutrient.mp.] AND [“apple.mp. or exp Malus/” OR “beet.mp. or Beta vulgaris/” OR “blueberry.mp. or exp Blueberry plants/” OR carob.mp. OR “carrot.mp. or exp Daucus carota/” OR “chickpea.mo. or exp Cicer/” OR “citrus.mp. or exp Citrus/” OR “cranberry.mp. or Vaccinium macrocarpon/” OR “kiwi.mp or exp Actinidia/” OR “pea.mp. or exp Peas/” OR “potato.mp or exp Solanum tuberosum/” OR chia.mp OR “flax.mp or exp Flax/” OR “hemp.mp. or Cannabis/” OR “corn.mp. or exp Zea mays/” OR “oat.mp. or exp Zea mays/” OR “rice.mp. or exp Oryza/” OR soy.mp. OR “wheat.mp or exp Triticum”]
Scopus	[“insoluble” AND “fiber”] AND [“phytochemical” OR “bioactive” OR “phytonutrient”] AND [“apple” OR “beet” OR “blueberry” OR “carob” OR “carrot” OR “chickpea” OR “citrus” OR “cranberry” OR “kiwi” OR “pea” OR “potato” OR “chia” OR “flax” OR “hemp” OR “corn” OR “oat” OR “rice” OR “soy” OR “wheat”]

**Table 2:** Fiber types, phenolic and flavonoid content, and antioxidant activity.

Insoluble Fiber Type Examined	Bioactive Content Measured	Plant Forms	Total Phenolic Content (TPC)		Antioxidant Activity (aa)	Article
				Total Flavenoid Content (TFC)		
GRAIN						
RICE	*n* = 5	stabilized rice bran	X free			Espinales et al. (2022) [16]
		germinated organic red rice	X	X	X	Nugraheni et al. (2022) [17]
		defatted rice bran	X	X	X	Zhao et al. (2018) [18]
		rice husk			X	Kuan et al. (2012) [19]
		KFSW and TK16 mature and immature rice grains	X	X	X	Lin et al. (2011) [20]
SOYBEAN	*n* = 1	Okara	X		X	Asghar et al. (2022) [21]
OAT	*n* = 1	oat milk byproduct	X	X	X	Wang (2023) [22]
TEFF	*n* = 1	teff	X free bound		X free bound	Caporizzi et al. (2023) [23]
CORN	*n* = 2	Corncob	X free, bound, esterified		X free, bound, esterified	Lau et al. (2019) [24]
		corncob nanofibers			X	Kuan et al. (2012) [19]
WHEAT	*n* = 2	durum and bread wheat flours	X		X	Cuidad-Mulero et al. (2020) [25]
		blue, black, and purple biofortified wheats	X free bound		X	Kumari et al. (2020) [26]
SEEDS/LEGUMES						
CAROB	*n* = 1	carob	X		X	Durazzo et al. (2014) [27]
CASHEW	*n* = 1	pomace	X	X	X	Medeiros et al. (2020) [28]
CHICKPEA	*n* = 1		X	X	X	Wang et al. (2023) [22]
LINSEED	*n* = 1	meal			X	Betrouche et al. (2022) [29]
LENTILS	*n* = 2	yellow, red, and green commercial and local lentils hull	X		X	Goncu et al. (2020) [30]
			X			Stefano et al. (2021) [31]
HEMP			X	X		Matilla et al. (2018) [32]
FRUITS/VEGETABLES						
MANGO	*n* = 3	mango peel, pulp, and seeds	X	X	X	Singh et al. (2016) [33]
			n/a			Sudha et al. (2015) [34]
			X		X	Abdul et al. (2012) [35]
TOMATO/PERSIMMON	*n* = 5	persimmon, tomato byproduct (seeds, peels, and pulp)	n/a			Diaz et al. (2020) [36]
			X		X	Chouaibi et al. (2019) [37]
			X		X	Isik et al. (2016) [38]
			X		X	Isik et al. (2016) [38]
					X	Betrouche et al. (2022) [29]
BEETS	*n* = 4	sugar beet pectin, beetroot leaf, beetroot	X	X	X	Singh et al. (2016) [33]
			X		X	Asadi et al. (2021) [39]
			n/a			Zagury et al. (2021) [40]
			X		X	Bainsal et al. (2021) [41]
APPLE	*n* = 4	pomace (seeds, peel, core)	X		X	Gouw et al. (2017) [42]
			X	X	X	Cerda-Tapia et al. (2015) [43]
			X		X	Rana et al. (2021) [44]
			X			Wang et al. (2019) [45]
SWISS CHARD	*n* = 1	leaves	n/a			Mzoughi et al. (2019) [46]
PEA	*n* = 1	butterfly pea and yellow pea	X		X	Singh et al. (2022) [47]
PEAR	*n* = 1	cladode	X			Saenz et al. (2012) [48]
ORANGE	*n* = 4	blood orange, kinnow, Khasi mandarin	X	X	X	Saikia et al. (2016) [49]
			X	X	X	Nagarajaiah et al. (2021) [50]
			X	X	X	Singh et al. (2016) [33]
			n/a			Russo et al. (2021) [51]
PINEAPPLE	*n* = 3	pomace, shell pomace				Larrauri et al. (1997) [52]
			X	X	X	Saikia et al. (2016) [49]
			X	X	X	Nagarajaiah et al (2021) [50]
GUAVA	*n* = 2	pomace	X	X	X	Medeiros et al. (2020) [28]
CHERRY	*n* = 2	pomace, sour cherry	X	X	X	Medeiros et al. (2020) [28]
			X	X	X	Gumul et al. (2020) [53]
PITAYA	*n* = 1	peel	X		X	Mai et al. (2022) [15]
CAPUASSU	*n* = 1	peel	X			Salgado et al. (2011) [54]
POMEGRANATE	*n* = 1	pomace	X	X	X	Singh et al. (2016) [33]
BANANA	*n* = 2	pomace, blossom of seeded banana	X	X	X	Saikia et al. (2016) [49]
			X	X	X	Singh et al. (2016) [33]
PLUM	*n* = 1	pomace	X	X	X	Singh et al. (2016) [33]
GRAPE	*n* = 3	pomace, Burmese grape, blue grape pomace	X	X	X	Saikia et al. (2016) [49]
			X	X	X	Nagarajaiah et al. (2021) [50]
			X	X	X	Singh et al. (2016) [33]
SAPODILLA	*n* = 1		X	X	X	Singh et al. (2016) [33]
EGGPLANT (BRINJAL)	*n* = 1		X	X	X	Singh et al(2016) [33]
CARROT	*n* = 1	orange carrot	X	X	X	Singh et al. (2016) [33]
GOURD	*n* = 1	bitter gourd	X	X	X	Singh et al. (2016) [33]
MENTHA	*n* = 1		X	X	X	Singh et al. (2016) [33]
GINSENG	*n* = 1	ginseng residue	X	X	X	Jiang et al. (2021) [55]
SPINACH	*n* = 1		X	X	X	Singh et al. (2016) [33]
BLUEBERRY	*n* = 1	pomace	X		X	Gouw et al. (2017) [42]
RASPBERRY	*n* = 1	red raspberry pomace	X		X	Gouw et al. (2017) [42]
CRANBERRY	*n* = 1	pomace	X		X	Gouw et al. (2017) [42]
LEMON	*n* = 1	sweet lemon	X	X	X	Nagarajaiah et al. (2021) [50]
CARAMBOLA	*n* = 1	pomace	X	X	X	Saikia et al. (2016) [49]
WATERMELON	*n* = 1	peel	X	X	X	Saikia et al. (2016) [49]
STINGING NETTLE	*n* = 1		n/a			Krawecka et al. (2021) [56]

An “X” indicates that for that insoluble fiber source, TPC, TFC, or AA were measured.

**Table 3:** Measurements of antioxidant activity (AA) utilized.

Measurement of AA	Definition
ABTS assay	free radical scavenging assay
DPPH assay	free radical scavenging assay
FRAP	ferric reducing antioxidant power assay
CAA	cellular antioxidant activity
TEAC	Trolox equivalent antioxidant assay
	beta-carotene bleaching assay
PCL	photochemiluminescence assay

**Table 4:** Specific bioactive compounds.

Bioactive Compound	IDF Fraction Found in	Form of IDF Fraction	Number of IDF Sources Bioactive WAS Found in	Sources
PHENOLIC ACIDS:				
FERULIC ACID	Rice	KFSW and TK16 mature and immature rice grains	*n* = 17	Lin et al. (2011) [20]
	corn	sweet corn cob		Lau et al. (2019) [24]
	Pineapple	pineapple pomace		Saikia et al. (2016) [49]
	grape	Burmese grape peel		Saikia et al. (2016) [49]
	carambola			Saikia et al. (2016) [49]
	banana	banana blossom		Saikia et al. (2016) [49]
	fava bean			Betrouche et al. (2022) [29]
	tomato	tomato byproduct		Betrouche et al. (2022) [29]
	mango	peel and pulp		Singh et al. (2016) [33]
	kinnow	peel and pulp		Singh et al. (2016) [33]
	banana	peel and pulp		Singh et al. (2016) [33]
	orange			Singh et al. (2016) [33]
	carrot	black carrot		Singh et al. (2016) [33]
	mango	mango pulp fiber waste (wet and dried)		Sudha et al. (2015) [34]
	ginseng	ginseng residue		Jiang et al. (2021) [55]
	cherry	sour cherry pomace		Gumul et al. (2020) [53]
	rice	defatted rice bran		Zhao et al. (2018) [18]
FERULIC ACID METHYL-ESTER	rice	defatted rice bran	*n* = 1	Zhao et al. (2018) [18]
CAFFEIC ACID	Rice	KFSW and TK16 mature and immature rice grains	*n* = 17	Lin et al. (2010) [20]
	pineapple	pineapple pomace		Saikia et al. (2016) [49]
	orange	Khasi mandarin orange peel		Saikia et al. (2016) [49]
	pomegranate	peel		Singh et al. (2016) [33]
	kinnow	peel and pulp		Singh et al. (2016) [33]
	mango	peel		Singh et al. (2016) [33]
	banana	peel and pulp		Singh et al. (2016) [33]
	grapes			Singh et al. (2016) [33]
	jambolana			Singh et al. (2016) [33]
	beetroot			Singh et al. (2016) [33]
	brinjal			Singh et al. (2016) [33]
	mentha			Singh et al. (2016) [33]
	bitter gourd			Singh et al. (2016) [33]
	carrot	black and orange		Singh et al. (2016) [33]
	mango	mango pulp fiber waste (wet and dried)		Sudha et al. (2015) [34]
	apple	Mexican apple		Cerdia-Tapia et al. (2015) [43]
	rice	defatted rice bran		Zhao et al. (2018) [18]
CAFFEIC ACID METHYL-ESTER	rice	defatted rice bran	*n* = 1	Zhao et al. (2018) [18]
CHLOROGENIC ACID	watermelon		*n* = 6	Saikia et al. (2016) [49]
	mango	mango pulp fiber waste (wet and dried)		Sudha et al. (2015) [34]
	apple	royal delicious, golden delicious, red delicious, red chief, red gold		Rana et al. (2021) [44]
	apple	Mexican apple		Cerdia-Tapia et al. (2015) [43]
	ginseng	ginseng residue		Jiang et al. (2021) [55]
	rice	defatted rice bran		Zhao et al. (2018) [18]
P-COUMARIC ACID	corn	sweet corn cob	*n* = 9	Lau et al. (2019) [24]
	pineapple	pineapple shell		Larrauri et al. (1997) [52]
	grape	Burmese grape peel		Saikia et al. (2016) [49]
	watermelon			Saikia et al. (2016) [49]
	fava bean			Betrouche et al. (2022) [29]
	tomato	tomato byproduct		Betrouche et al. (2022) [29]
	mango	mango pulp fiber waste (wet and dried)		Sudha et al. (2015) [34]
	ginseng	ginseng residue		Jiang et al. (2021) [55]
	rice	defatted rice bran		Zhao et al. (2018) [18]
GALLIC ACID	pineapple	pineapple pomace	*n* = 18	Saikia et al. (2016) [49]
	grape	Burmese grape peel		Saikia et al. (2016) [49]
	orange	Khasi mandarin peel		Saikia et al. (2016) [49]
	carambola			Saikia et al. (2016) [49]
	watermelon			Saikia et al. (2016) [49]
	banana	banana blossom		Saikia et al. (2016) [49]
	banana	peel and pulp		Singh et al. (2016) [33]
	jambolan	peel and pulp		Singh et al. (2016) [33]
	pomegranate	peel and pulp		Singh et al. (2016) [33]
	mango	peel and pulp		Singh et al. (2016) [33]
	sapodilla			Singh et al. (2016) [33]
	grapes			Singh et al. (2016) [33]
	beetroot			Singh et al. (2016) [33]
	bitter gourd			Singh et al. (2016) [33]
	mentha			Singh et al. (2016) [33]
	carrot	black carrot		Singh et al. (2016) [33]
	mango	mango pulp fiber waste (wet and dried)		Sudha et al. (2015) [34]
	rice	defatted rice bran		Zhao et al. (2018) [18]
SYRINGIC ACID	fava bean		*n* = 8	Betrouche et al. (2022) [29]
	tomato	tomato byproduct		Betrouche et al. (2022) [29]
	pineapple	pineapple shell		Saikia et al. (2016) [49]
	orange	Khasi mandarin peel		Saikia et al. (2016) [49]
	watermelon			Saikia et al. (2016) [49]
	banana	banana blossom		Saikia et al. (2016) [49]
	ginseng	ginseng residue		Jiang et al. (2021) [55]
	rice	defatted rice bran		Zhao et al. (2018) [55]
TRANS CINNAMIC ACID	pineapple	pineapple shell	*n* = 2	Larrauri et al. (1997) [52]
	mango	mango pulp fiber waste		Sudha et al. (2015) [34]
CINNAMIC ACID	ginseng	ginseng residue	*n* = 1	Jiang et al. (2021) [55]
SINAPIC ACID	pomegranate	peel and pulp	*n* = 4	Singh et al. (2016) [33]
	kinnow	peel and pulp		Singh et al. (2016) [33]
	grapes			Singh et al. (2016) [33]
	jambolan			Singh et al. (2016) [33]
4-HYDROXYBENZOIC ACID	fava bean		*n* = 3	Betrouche et al. (2022) [29]
	tomato	tomato byproduct		Betrouche et al. (2022) [29]
	rice	defatted rice bran		Zhao et al. (20118) [18]
SALICYLIC ACID	pineapple	pineapple shell	*n* = 4	Larrauri et al. (1997) [52]
	acerola	cherry		Medeiros et al. (2020) [28]
	guava			Medeiros et al. (2020) [28]
	cashew			Medeiros et al. (2020) [28]
VANILLIN	rice	defatted rice bran	*n* = 1	Zhao et al. (2018) [18]
VANILLIC ACID	acerola	cherry	*n* = 4	Medeiros et al. (2020) [28]
	guava			Medeiros et al. (2020) [28]
	cashew			Medeiros et al. (2020) [28]
	Rice	defatted rice bran		Zhao et al. (2018) [18]
FLAVONOIDS:				
FLAVANOLS				
QUERCETIN	carambola		*n* = 19	Saikia et al. (2016) [49]
	grape	Burmese grape peel		Saikia et al. (2016) [49]
	banana	banana blossom		Saikia et al. (2016) [49]
	tomato	tomato byproduct		Betrouche et al. (2022) [29]
	pomegranate	pulp		Singh et al. (2016) [33]
	mango	peel and pulp		Singh et al. (2016) [33]
	banana	peel		Singh et al. (2016) [33]
	sapodilla	peel and pulp		Singh et al. (2016) [33]
	jambolan			Singh et al. (2016) [33]
	grapes			Singh et al. (2016) [33]
	beetroot			Singh et al. (2016) [33]
	carrot	black carrot		Singh et al. (2016) [33]
	spinach			Singh et al. (2016) [33]
	acerola	cherry		Medeiros et al. (2020) [28]
	guava			Medeiros et al. (2020) [28]
	cashew			Medeiros et al. (2020) [28]
	apple	royal delicious, golden delicious, red delicious, red chief, red gold		Rana et al. (2021) [44]
	apple	Mexican apple		Cerdia-Tapia et al. (2015) [43]
	rice	defatted rice bran		Zhao et al. (2018) [18]
QUERCITRIN	apple	royal delicious, golden delicious, red delicious, red chief, red gold	*n* = 2	Rana et al. (2021) [44]
	apple	Mexican apple		Cerdia-Tapia et al. (2015) [43]
QUERCETIN DERIVATIVE	fava bean		*n* = 2	Betrouche et al. (2022) [29]
	tomato	tomato byproduct		
ISOQUERCITRIN	ginseng	ginseng residue	*n* = 3	Jiang et al. (2021) [55]
	rice	defatted rice bran		Zhao et al. (2018) [18]
	apple	royal delicious, golden delicious, red delicious, red chief, red gold		Rana et al. (2021) [44]
MYRICETIN	pineapple	pineapple shell	*n* = 4	Larrauri et al. (1997) [52]
	acerola	cherry		Medeiros et al. (2020) [28]
	guava			Medeiros et al. (2020) [28]
	cashew			Medeiros et al. (2020) [28]
KAEMPFEROL	kinnow	pulp	*n* = 9	Singh et al. (2016) [33]
	mango	peel and pulp		Singh et al. (2016) [33]
	banana	peel		Singh et al. (2016) [33]
	sapodilla	peel and pulp		Singh et al. (2016) [33]
	jambolan			Singh et al. (2016) [33]
	grapes			Singh et al. (2016) [33]
	beetroot			Singh et al. (2016) [33]
	carrot	black carrot		Singh et al. (2016) [33]
	spinach			Singh et al. (2016) [33]
RUTIN	tomato	tomato byproduct	*n* = 3	Betrouche et al. (2022) [29]
	apple	Mexican apple		Cerdia-Tapia et al. (2015) [43]
	cherry	sour cherry pomace		Gumul et al. (2020) [53]
RUTIN HYDRATE	orange	Khasi mandarin	*n* = 3	Saikia et al. (2016) [49]
	carambola			Saikia et al. (2016) [49]
	banana	banana blossom		Saikia et al. (2016) [49]
AVICULARIN	apple	Mexican apple	*n* = 1	Cerdia-Tapia et al. (2015) [43]
HYPERIN	apple	Mexican apple	*n* = 1	Cerdia-Tapia et al. (2015) [43]
FLAVAN-3-OLS				
CATECHIN	grape	Burmese grape peel	*n* = 13	Saikia et al. (2016) [49]
	carambola			Saikia et al. (2016) [49]
	watermelon			Saikia et al. (2016) [49]
	fava bean			Betrouche et al. (2022) [29]
	pomegranate	peel and pulp		Singh et al. (2016) [33]
	mango	peel and pulp		Singh et al. (2016) [33]
	banana	peel and pulp		Singh et al. (2016) [33]
	sapodilla	peel and pulp		Singh et al. (2016) [33]
	bitter gourd			Singh et al. (2016) [33]
	grapes	whole		Singh et al. (2016) [33]
	mango	mango pulp fiber waste (wet and dried)		Sudha et al. (2015) [34]
	ginseng	ginseng residue		Jiang et al. (2021) [55]
	rice	defatted rice bran		Zhao et al. (2018) [18]
EPICATECHIN	mango	mango pulp fiber waste (wet and dried)	*n* = 5	Sudha et al. (2015) [34]
	apple	royal delicious, golden delicious, red delicious		Rana et al. (2021) [44]
	apple	Mexican apple		Cerdia-Tapia et al. (2015) [43]
	ginseng	ginseng residue		Jiang et al. (2021) [55]
	rice	defatted rice bran		Zhao et al. (2018) [18]
PROTOCATECHUIC ACID	rice	defatted rice bran	*n* = 1	Zhao et al. (2018) [18]
PYROCATECHINIC ACID	fava bean		*n* = 4	Betrouche et al. (2022) [29]
	tomato	tomato byproduct		Betrouche et al. (2022) [29]
	kinnow	peel		Singh et al. (2016) [33]
	banana	pulp		Singh et al. (2016) [33]
ANTHOCYANINS				
TOTAL ANTHOCYANINS	wheat	durum and bread wheat flour	*n* = 4	Cuidad-Murelo et al. (2020) [25]
	wheat	black, blue, and purple		Kumari et al. (2020) [26]
	mango	green peel, green pulp, ripe peel and ripe pulp flour		Abdul et al. (2015) [35]
	cherry	sour cherry pomace		Gumul et al. (2020) [53]
FLAVANONES				
NARINGENIN	tomato	tomato byproduct	*n* = 1	Betrouche et al. (2022) [29]
HESPERITIN	acerola	cherry	*n* = 3	Medeiros et al. (2020) [28]
	guava			Medeiros et al. (2020) [28]
	cashew			Medeiros et al. (2020) [28]
FLAVONES				
APIGENIN	tomato	tomato byproduct	*n* = 1	Betrouche et al. (2022) [29]
CHRYSIN	acerola	cherry	*n* = 3	Medeiros et al. (2020) [28]
	guava			Medeiros et al. (2020) [28]
	cashew			Medeiros et al. (2020) [28]
OTHER				
PHLORIDZIN	apple	royal delicious, golden delicious, red delicious, red chief, red gold	*n* = 2	Rana et al. (2021) [44]
	apple	Mexican apple		Cerdia-Tapia et al. (2015) [43]
NON-FLAVONOID COMPOUNDS:				
TANNINS				
TANNIC ACID	pineapple	pineapple shell	*n* = 1	Larrauri et al. (1997) [52]
TOTAL TANNINS	pineapple		*n* = 5	Nagarajaiah et al. (2021) [50]
	lemon	sweet lemon		Nagarajaiah et al. (2021) [50]
	grapes	blue grapes		Nagarajaiah et al. (2021) [50]
	orange			Nagarajaiah et al. (2021) [50].
	capuassu	capuassu peel		Salgado et al. (2011) [54]
STILLBENES				
RESVERATROL	rice	rice flour	*n* = 10	Betrouche et al. (2022) [29]
	fava bean			Betrouche et al. (2022) [29]
	linseed	linseed meal		Betrouche et al. (2022) [29]
	pomegranate	pulp		Singh et al. (2016) [33]
	banana	peel and pulp		Singh et al. (2016) [33]
	grapes			Singh et al. (2016) [33]
	sapodilla	peel		Singh et al. (2016) [33]
	spinach			Singh et al. (2016) [33]
	mentha			Singh et al. (2016) [33]
	ginseng	ginseng residue		Jiang et al. (2021) [55]
RESVERATROL DERIVATIVE	rice	rice flour	*n* = 3	Betrouche et al. (2022) [29]
	fava bean			
	linseed	linseed meal		
TOTAL STILLBENES	rice	rice flour	*n* = 3	Betrouche et al. (2022) [29]
	fava bean			
	linseed	linseed meal		
TOCOPHEROLS AND TOCOTRIENOLS				
ALPHA-TOCOPHEROL	Rice	KFSW and TK16 mature and immature rice grains	*n* = 2	Lin et al. (2011) [20]
	wheat	durum and bread wheat flour		Cuidad-Murelo et al. (2020) [25]
BETA-TOCOPHEROL	Rice	KFSW and TK16 mature and immature rice grains	*n* = 2	Lin et al. (2011) [20]
	wheat	durum and bread wheat flour		Cuidad-Murelo et al. (2020) [25]
GAMMA-TOCOPHEROL	Rice	KFSW and TK16 mature and immature rice grains	*n* = 1	Lin et al. (2011) [20]
DELTA-TOCOPHEROL	Rice	KFSW and TK16 mature and immature rice grains	*n* = 1	Lin et al. (2011) [20]
ALPHA-TOCOTRIENOL	Rice	KFSW and TK16 mature and immature rice grains	*n* = 1	Lin et al. (2011) [20]
BETA-TOCOTRIENOL	Rice	KFSW and TK16 mature and immature rice grains	*n* = 1	Lin et al. (2011) [20]
GAMMA-TOCOTRIENOL	Rice	KFSW and TK16 mature and immature rice grains	*n* = 1	Lin et al. (2011) [20]
VITAMIN C				
ASCORBIC ACID	lemon	sweet lemon	*n* = 5	Nagarajaiah et al. (2021) [50]
	grapes	blue grapes		Nagarajaiah et al. (2021) [50]
	orange			Nagarajaiah et al. (2021) [50]
	pineapple			Nagarajaiah et al. (2021) [50]
	mango	green peel, green pulp, ripe peel and ripe pulp flour		Abdul et al. (2012) [35]
RETINOL EQUIVALENTS	persimmon	destringed	*n* = 1	Diaz et al. (2020) [36]
CAROTENOIDS				
TOTAL CAROTENOIDS	stinging nettle		*n* = 10	Krawecka et al. (2021) [56]
	lemon	sweet lemon		Nagarajaiah et al. (2021) [50]
	pineapple			Nagarajaiah et al (2021) [50]
	orange			Nagarajaiah et al. (2021) [50]
	rice	rice flour		Betrouche et al. (2022) [29]
	fava bean			Betrouche et al. (2022) [29]
	tomato	tomato byproduct		Betrouche et al. (2022) [29]
	linseed	linseed meal		Betrouche et al. (2022) [29]
	persimmon	destringed		Diaz et al. (2020) [36]
	mango	green peel, green pulp, ripe peel and ripe pulp flour		Abdul et al. (2012) [35]
BETA-CAROTENE	corn	sweet corn cob	*n* = 7	Lau et al. (2019) [24]
	rice	rice flour		Betrouche et al. (2022) [29]
	fava bean			Betrouche et al. (2022) [29]
	tomato	tomato byproduct		Betrouche et al. (2022) [29]
	linseed	linseed meal		Betrouche et al. (2022) [29]
	persimmon	destringed		Diaz et al. (2020) [36]
	mango	mango pulp fiber waste (wet and dried)		Sudha et al. (2015) [34]
BETA-CRYPTOXANTHIN	fava bean		*n* = 4	Betrouche et al. (2022) [29]
	tomato	tomato byproduct		Betrouche et al. (2022) [29]
	linseed	linseed meal		Betrouche et al. (2022) [29]
	persimmon	destringed		Diaz et al. (2020) [36]
NEOXANTHIN	persimmon	destringed	*n* = 1	Diaz et al. (2020) [36]
VIOLAXANTHIN	persimmon	destringed	*n* = 1	Diaz et al. (2020) [36]
ZEAXANTHIN	corn	sweet corn cob	*n* = 5	Lau et al. (2019) [24]
	rice	rice flour		Betrouche et al. (2022) [29]
	fava bean			Betrouche et al. (2022) [29]
	tomato	tomato byproduct		Betrouche et al. (2022) [29]
	linseed	linseed meal		Betrouche et al. (2022) [29]
LUTEIN	corn	sweet corn cob	*n* = 5	Lau et al. (2019) [24]
	fava bean			Betrouche et al. (2022) [29]
	tomato	tomato byproduct		Betrouche et al. (2022) [29]
	linseed	linseed meal		Betrouche et al. (2022) [29]
	mango	mango pulp fiber waste (wet and dried)		Sudha et al. (2015) [34]
LYCOPENE	rice	rice flour	*n* = 5	Betrouche et al. (2022) [29]
	fava bean			Betrouche et al. (2022) [29]
	tomato	tomato byproduct		Betrouche et al. (2022) [29]
	linseed	linseed meal		Betrouche et al. (2022) [29]
	persimmon	destringed		Diaz et al. (2020) [36]
CHLOROPHYLLS				
CHLOROPHYLL A	stinging nettle		*n* = 1	Krawecka et al. (2021) [56]
CHLOROPHYLL B	stinging nettle		*n* = 1	Krawecka et al. (2021) [56]
BETALAINS				
BETACYANIN	pitaya	pitaya peel powder	*n* = 1	Mai et al. (2022) [15]
OTHER				
TYROSOL	fava bean		*n* = 1	Betrouche et al. (2022) [29]
	tomato	tomato byproduct	*n* = 1	Betrouche et al. (2022) [29]
γ-ORYZANOL	Rice	KFSW and TK16 mature and immature rice grains	*n* = 2	Lin et al. (2011) [20]
	rice	stabilized rice bran		Espinales et al. (2022) [16]
BETA-GLUCAN	rice	stabilized rice bran	*n* = 2	Espinales et al. (2022) [16]
	oat	oat milk byproduct		Wang et al. (2023) [22]
γ-AMINOBUTYRIC ACID (GABA)	Rice	stabilized rice bran	*n* = 1	Espinales et al. (2022) [16]

## Appendix A. Excluded Article Bibliography at Full-Text Screen

Reasons for exclusion:

- Not originally published in English (*n* = 1) (Delgado-Nieblas, et al. [62]);
- Extracted insoluble fiber but only examined the antioxidant effects/phytochemical; content of the soluble fiber (*n* = 1) (Multari, et al. [63]);
- Focused on effect of food processing (*n* = 2) (Chen, et al. [64] and Garcia, et al. [65]);
- Did not measure outcomes relevant to this article (*n* = 3);
  ⚬ Surface tension and solvent diffusional properties (Verdú, et al. [66]);
  ⚬ Effect of enzymatic pretreatment of fruit pomaces (Alberici, et al. [67]);
    ■ Could be valuable source in the future research section, if fortification is going to occur how can we optimize/maintain the nutritive quality of the extracted IDF;
  ⚬ Created an extract that did not have IDF in it (Cairone, et al. [68]);
  ⚬ No discussion/testing of phytochemicals (Liu, et al. [69]).
